# Effect of dopamine partial agonist antipsychotics on suicide risk in persons with schizophrenia and schizoaffective disorders: A systematic review and meta-analysis

**DOI:** 10.1016/j.schres.2026.09.006

**Published:** 2026-09-08

**Authors:** Jeff W. Jin, Charlotte J. Winkler, Heather B. Blunt, Natalie B. Riblet

**Affiliations:** a Dartmouth-Hitchcock Medical Center – Department of Psychiatry, USA; b Biomedical Libraries, Dartmouth College, Hanover, NH, USA; c Mental Health, White River Junction VA Healthcare System, White River Junction, VT, 05009, USA; d Psychiatry, Geisel School of Medicine at Dartmouth College, Hanover, NH, 03755, USA; e Dartmouth Institute, Geisel School of Medicine at Dartmouth College, Hanover, NH, 03755, USA

**Keywords:** Schizophrenia, Schizoaffective, Dopamine partial agonist, Antipsychotic, Suicide

## Abstract

**Background and hypothesis:**

Clozapine is the only antipsychotic with protective effects against suicide in schizophrenia (SCZ). Newer dopamine partial agonist (DPA) antipsychotics have better tolerability and modulate serotonin, dopamine, and *N*-methyl-d-aspartate neurotransmission pathways implicated in suicide. We aim to investigate the effects of DPAs on suicide in SCZ.

**Methods:**

We searched seven databases up to February 2026 for SCZ studies that reported suicide data. The primary outcome was suicide deaths and attempts; suicidal ideation was added as a secondary outcome. Random effects meta-analyses quantified suicide risk in randomized controlled trials (RCT) while single proportion meta-analyses assessed longitudinal suicide risk in open label extension trials (OLE). For RCTs, sensitivity analyses were conducted and subgroup analyses explored the impact of dose, drug type, and comparator arm.

**Study results:**

Twenty articles were included; thirteen excluded higher suicide risk participants. Compared to placebo control, DPAs did not significantly change the risk of primary [RR = 0.65, *p* = 0.38] or secondary [RR = 0.63, *p* = 0.15] suicide outcomes. Subgroup and sensitivity analyses were not statistically significant. For OLEs, there was a significant increase in the incidence of primary [Ip = 0.004, *p* = 0.048] and secondary [Ip = 0.024, *p* = 0.0013] suicide outcomes, but there was marked study heterogeneity.

**Conclusion:**

There is no current trial evidence to show that DPAs significantly impact suicide outcomes in SCZ. The signal from OLEs should be interpreted cautiously due to heterogeneity and requires replication. An effective clozapine alternative is needed for suicide prevention in SCZ.

## 1. Introduction

Schizophrenia spectrum disorders (SCZ) primarily include schizophreniform, schizophrenia, and schizoaffective disorders and have an estimated lifetime prevalence of 1% (Rössler et al., 2005; Saha et al., 2005). SCZ is a significant contributor to the global burden of disease and has increased in burden contribution over time (Murray and Lopez, 1997; Solmi et al., 2023). In the United States in 2024, the estimated societal burden of SCZ was $366.8 billion with substantial indirect costs from premature mortality (Krasa et al., 2026). There is a substantial mortality gap between persons with schizophrenia and the general population (Saha et al., 2007). Reductions in life expectancy in SCZ are estimated between 15 and 20 years with high contributions from cardiometabolic disease and suicide (Laursen, 2011; Saha et al., 2007).

Suicide is a leading cause of premature death in SCZ (Olfson et al., 2015). The lifetime world prevalence of suicide attempts in SCZ is 26.8% with a significant increased prevalence in North America (35.9%) and Europe and Central Asia (32.2%) (Lu et al., 2020). Around 4–10% of persons with SCZ will die by suicide during their lifetimes with male sex, younger age, and higher intelligence as significant risk factors (Cassidy et al., 2017; Keepers et al., 2020; Palmer et al., 2005). More broadly, a stress-diathesis model of suicidal behavior has been proposed with additional neurobiological evidence implicating serotonergic, dopaminergic, and glutamatergic systems (Mann, 2003; van Heeringen and Mann, 2014). Alterations in cerebrospinal fluid serotonin metabolites and post-mortem serotonin neurotransmission were observed in patients with suicidal behavior (Carballo et al., 2008; Ryding et al., 2008). Additionally, dysregulation in striatal dopamine receptor binding and predictability of suicidal ideation from reduced dorsal striatal gray matter volume have also implicated dopaminergic dysfunction, a hypothesized etiology of SCZ (Fitzgerald et al., 2017; Ho et al., 2018; Howes and Kapur, 2009). Furthermore, *N*-methyl-d-aspartate (NMDA) receptor alterations observed in the frontal cortex and hippocampus in suicide victims suggest implications of NMDA dysfunction in suicide (Nowak et al., 1995; Sowa-Kućma et al., 2013).

Despite high occurrence of suicide in SCZ, pharmacologic interventions specifically targeting suicide remain very limited. Clozapine is the only antipsychotic with protective effects against suicide in SCZ (Hennen and Baldessarini, 2005; Meltzer et al., 2003). Additionally, clozapine demonstrates superior efficacy in the reduction of positive and negative symptoms, overall psychopathology, and relapse rates in SCZ compared to first and second-generation antipsychotics (Wagner et al., 2021). However, the efficacy of clozapine is countered by shifting monitoring protocols in the United States and significant side effects including neutropenia, constipation, hypotension, sedation, and weight gain that pose additional barriers to existing adherence difficulties for persons with SCZ (Iqbal et al., 2020; Meyer and Rubio, 2025; Verdoux et al., 2025).

Dopamine partial agonists (DPA) are a newer class of antipsychotics characterized by their unique modulation of dopamine through partial agonism of D2 and D3 receptors and primarily include aripiprazole, oxaripiprazole, brexpiprazole, cariprazine, and lumateperone (Chen et al., 2022; Keks et al., 2020; McCutcheon et al., 2023). Compared to other antipsychotics, DPAs have increased tolerability due to lower risks of weight gain, hyperprolactinemia, sedation, and anticholinergic side effects which are associated with low discontinuation rates (Keks et al., 2020). Contrary to clozapine, DPA-associated lab monitoring is much less rigorous and follows standard antipsychotic monitoring guidelines.

DPAs also modulate neurotransmission through pathways implicated in suicide – serotonergic, dopaminergic, and glutamatergic. In addition to D2 and D3 partial agonism, several DPAs also modulate serotonin activity through potent serotonin transporter (SERT) inhibition, 5-hydroxytryptamine (5-HT) 1A receptor partial agonism, and 5-HT2A and 2B receptor antagonism (Diefenderfer and Iuppa, 2017; Stahl, 2016; Vanover et al., 2019). Lumateperone enhances NMDA receptor function and indirectly modulates glutamine neurotransmission (Snyder et al., 2015). Additionally, DPAs have clinical antimanic and antidepressant activity with demonstrated reduction of impulsivity, a key component of the proposed stress-diathesis model of suicidal behavior, in animal models of mania (Milienne-Petiot et al., 2017; Taylor et al., 2023). Furthermore, DPAs are associated with lower negative symptom severity compared to high affinity D2 antagonists and may have potential benefit for secondary negative and cognitive symptoms in schizophrenia (Correll and Schooler, 2020; de Beer et al., 2024). These characteristics suggest that DPAs may be promising candidates for pharmacologic suicide prevention.

Suicide in SCZ has considerable negative burdens on quality of life, financial costs, and premature mortality. Pharmacologic interventions for suicide in SCZ are limited to clozapine, an effective antipsychotic with notable burdens of lab monitoring and side effects. There is a clear need for a more benign antipsychotic alternative with protective effects against suicide. DPAs may fill this gap because of pharmacologic activity in neurotransmitter pathways implicated in suicidal behavior. Given this, we conducted a systematic review and meta-analysis to determine the effects of DPAs on suicide risk in persons with SCZ. We hypothesize that there will be a small, significant protective effect of DPAs on suicidal attempts, behaviors, and ideation in persons with SCZ compared to placebo control. Secondarily, we hypothesize that there will be differences in suicidal risk in persons with SCZ taking DPAs enrolled in open-label studies with longer safety monitoring duration.

## 2. Methods

For the current study, we registered the protocol (CRD42023449336) with the International Prospective Register of Systematic Reviews (PROSPERO) prior to conducting the final data analysis and followed the Preferred Reporting Items for Systematic Reviews and Meta-Analyses (PRISMA) guidelines (Page et al., 2021; Rethlefsen et al., 2021).

### 2.1. Search strategy

We conducted a search of PubMed, MEDLINE, PsycINFO, Scopus, Cochrane Central Register of Controlled Trials, Europe PMC, and ClinicalTrials.gov databases from inception up to February 2026. The search strategy focused on medical subject headings (MeSH) and variations of keywords related to: (“Schizophrenia”) AND (“Suicide”) AND (“Antipsychotic”) AND (“Clinical Trial”). The search strategy was designed and peer-reviewed by two independent librarians and search terms were adapted to respective databases. Please refer to the supplementary materials for further detailed information on search strategy.

### 2.2. Inclusion and exclusion criteria

The major inclusion criteria were: [1] publication in English language, [2] studies of adult human subjects, [3] randomized controlled trials (RCT) or open-label extension studies (OLE) of completed randomized controlled trials, [4] inclusion of individuals with Diagnostic and Statistical Manual of Mental Disorders, Fourth Edition (DSM-IV), or revised diagnosis of SCZ (schizophrenia, schizoaffective disorder, schizophreniform disorder), [5] intervention of dopamine partial agonist antipsychotic agents including brexpiprazole, cariprazine, lumateperone, oxaripiprazole, and other D2 or D3 partial agonists, and [6] reporting of outcomes or suicide deaths, attempts, or ideation. Suicidal behavior was accepted as part of our definition of suicide attempt and studies reporting this outcome were included accordingly.

The notable exclusion criteria were: [1] non-SCZ conditions or diagnoses including subclinical or prodromal states (early phase psychosis, clinical high-risk for psychosis), substance-induced psychosis, delusional disorder, or schizotypal personality disorder, [2] non-DPA interventions and aripiprazole, [3] lack of reported suicide outcomes (deaths, attempts, or ideation) or studies where data on suicide outcomes were not able to be obtained, and [4] open-label trials that were not extensions of or did not include participants from prior RCTs. Studies on aripiprazole were excluded to focus on newer dopamine partial agonist antipsychotics with regulatory approval dates by the Food and Drug Administration (FDA) after 2013. Oxaripiprazole was a novel investigational antipsychotic during this time, so related studies were included despite lack of FDA approval. Articles without specific report of suicide outcomes measured with a validated instrument were excluded if data on suicide outcomes were not obtainable from supplementary materials, results from clinical trials registries, or contact with the corresponding author or listed trial contact or sponsor.

### 2.3. Screening and data extraction

Two reviewers (JJ, NR) independently screened the search results by title, abstract, full manuscript (where indicated), and eligibility using Rayyan QCRI software (Ouzzani et al., 2016). Disagreements were resolved through discussion and consensus agreement. Two independent reviewers (JJ, CW) extracted data from eligible studies for formal analysis using a standardized data collection form.

### 2.4. Quality assessment

Two reviewers (JJ, NR) independently assessed study quality and bias risk assessment with the revised Cochrane Risk of Bias Tool (RoB 2) across five domains: randomization, intervention assignment and adherence, missing outcome data, outcome measurement, and selection or reported results (Sterne et al., 2019). The overall bias risk for all studies was categorized as low risk, some bias concerns, or high risk. Disagreements were resolved through discussion and consensus agreement.

### 2.5. Outcomes

Primary outcomes included suicide deaths and attempts. We chose these measures since they are the most robust measures of suicide risk. Secondary outcomes included suicide deaths, attempts, and ideation. Suicidal ideation was included as a secondary outcome as suicide deaths and attempts are a rare occurrence that may be further limited by the short-term duration of randomized controlled trials and exclusion of participants with higher suicide risk.

### 2.6. Data synthesis and analysis

We conducted random effects meta-analyses for RCTs and single proportion meta-analyses for single-arm OLEs of suicide outcomes using Stata SE18 software. Statistical significance was measured at *p* < 0.05. Risk ratios (RR) and 95% confidence intervals (95% CI) were calculated to estimate intervention effect size. For study arms with zero events, a continuity correction of 0.5 was added to prevent mathematical impossibility (Bradburn et al., 2007; J. Sweeting et al., 2004). The Peto Odds Ratio was considered due to the presence of arms with zero outcome events but was ultimately not used because of the risks of effect overestimation due to unbalanced group sizes (Brockhaus et al., 2016). In the case of an meta-analysis of rare events, it is recommended that all studies contribute to the analysis regardless of zero or non-zero status events ((Böhning et al., 2015). To account for many zero values and unbalanced group sizes in placebo-controlled trials, we conducted a sensitivity analysis with Poisson or negative binomial regression using the number of patient years as an offset. We calculated an incidence rate ratio (IRR) with 95% confidence interval. We tested the goodness of fit of the data prior to conducting the analysis and used Poisson or negative binomial as appropriate.

Heterogeneity was measured using *I*^2^, *H*^2^, and Cochran Q test statistics. Heterogeneity was defined as an *I*^2^ > 50% and a *p* < 0.1. Meta-analysis results were presented in forest plots. Publications bias was assessed with funnel plots and Egger’s tests. For RCTs, sensitivity analyses were conducted to exclude studies classified as having “some concerns” or “high risk” of bias. Additionally, subgroups analyses were conducted to explore the impact of antipsychotic agent and choice of comparator arm. Due to too few studies and multiple zero values across available studies, subgroup analysis of suicide attempts and deaths by antipsychotic agent was deferred due to limited utility of meta-analysis or regression sensitivity analysis. A subgroup analysis by antipsychotic dose was similarly deferred. These data were presented qualitatively instead.

## 3. Results

### 3.1. Search results and quality assessment

The initial search resulted in 5074 articles and 3558 articles remained after removal of 1516 duplicates. After the exclusion of 3206 articles from abstract screening, 352 articles were retrieved for full review. After article review, 20 articles met inclusion criteria (Supplementary Fig. S1) including 15 RCTs (*N* = 5700) – five brexpiprazole (*N* = 2260), seven cariprazine (*N* = 2703), two lumateperone (*N* = 701), and one oxaripiprazole (*N* = 36). Five OLEs (*N* = 2200) also met inclusion criteria – three brexpiprazole (*N* = 1521) and two cariprazine (*N* = 679).

Detailed characteristics of the included studies are summarized in Table 1. Study samples ranged from 32 to 729 participants for RCTs and 93 to 1031 participants for OLEs. Brexpiprazole and cariprazine were the most studied DPAs. 11 RCTs had a study duration of 6 weeks or less while all OLEs have trial periods of around 1 year. The Columbia Suicide Severity Rating Scale (C-SSRS) was the most common method of suicide assessment; 13 studies (9 RCTs and 4 OLEs) excluded participants with increased risk of suicide. For comparator arms in RCTs, 14 RCTs used a placebo control condition while two of these RCTs had additional aripiprazole and quetiapine arms. One RCT used risperidone without a control condition. Quality assessment showed 13 RCTs with low bias risk and two RCTs with some concerns of bias. All five OLEs had some concerns of bias. Risk of bias assessments is summarized in supplementary materials (Figs. S2, S3; Tables S1, S2).

### 3.2. Meta-analysis of randomized controlled trials

**Fig. 1.**
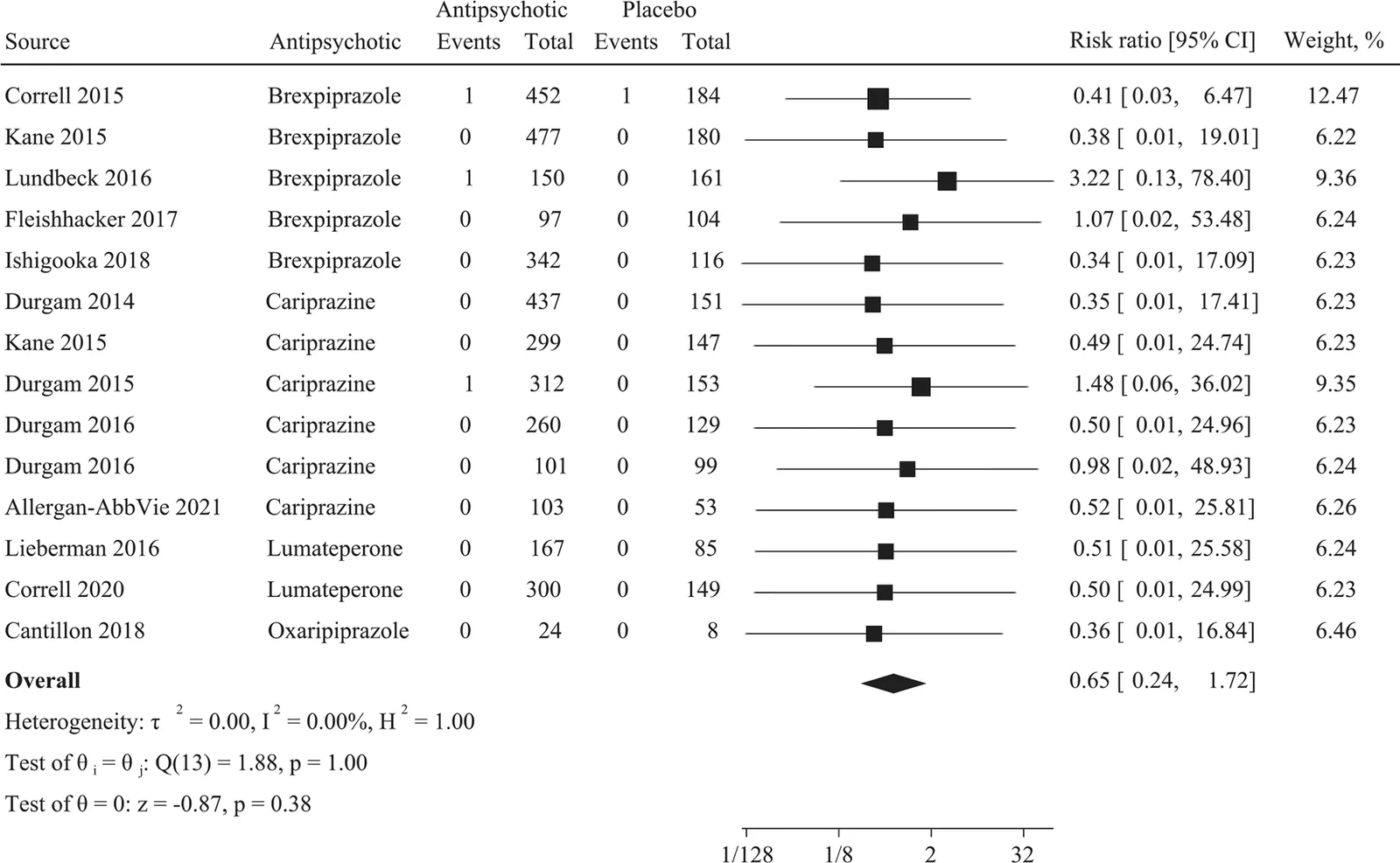
Meta-analysis of suicide deaths and attempts in randomized controlled trials of dopamine partial agonists.

**Fig. 2.**
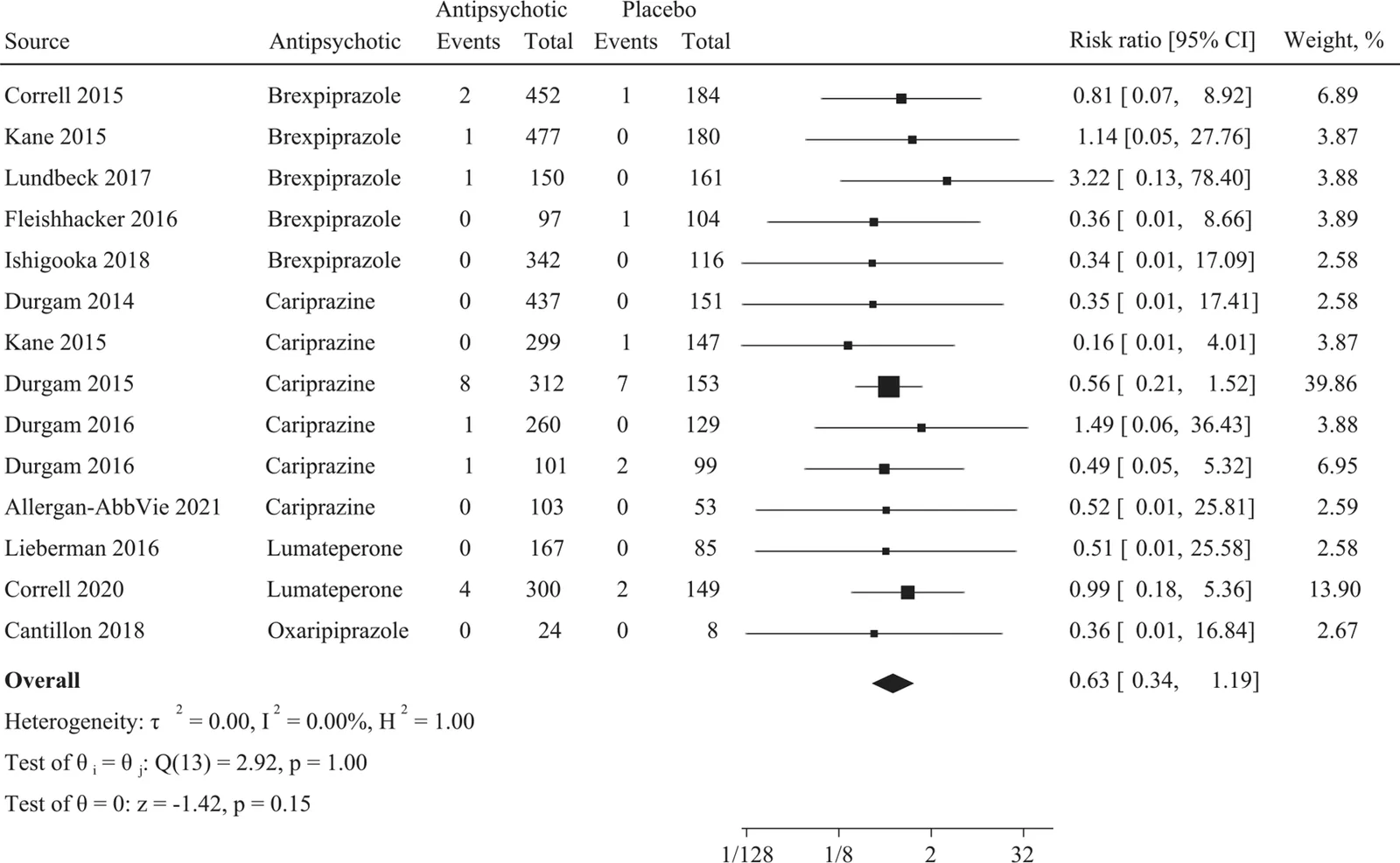
Meta-analysis of suicide deaths, attempts, and ideation in randomized controlled trials of dopamine partial agonists.

Of the 14 RCTs comparing DPAs to placebo control, there was no significant change in the risk of suicide deaths or attempts [RR = 0.65, 95% CI = 0.24–1.72, *p* = 0.38] and low concern for heterogeneity [*I*^2^ = 0%, *H*^2^ = 1; Q(13) = 1.88, *p* = 1] (Fig. 1). There was also no significant change in the risk of suicide deaths, attempts, or ideation [RR = 0.63, 95% CI = 0.34–1.19, *p* = 0.15; IRR: 0.76, 95% CI = 0.20–2.96, *p* = 0.69] and low concern for heterogeneity [*I*^2^ = 0%, *H*^2^ = 1; Q(13) = 2.92, *p* = 1] (Fig. 2). Sensitivity analyses of both primary and secondary suicide outcomes did not yield statistically different results (Supplementary Figs. S4, S5). Finally, while the incidence of suicide deaths or attempts was higher among DPAs relative to control (14 RCTs, IRR 1.67, 95% CI: 0.17–16.33, *p* = 0.66), the CI around the estimate was very wide and results were not significant (Table S3).

Formal subgroups analyses by antipsychotic agent (brexpiprazole, cariprazine) and choice of comparator arm (alternative antipsychotics of aripiprazole, quetiapine, and risperidone) did not reveal significant changes in primary or secondary suicide outcomes (Supplementary Figs. S6, S8, S10). There were low concerns for heterogeneity across all subgroup analyses. Sensitivity analyses showed no additional changes in significance (Supplementary Figs. S7, S9, S11). Further details on subgroup analyses and qualitative analysis by antipsychotic dose are provided in the supplementary materials (Supplementary Materials, Table S4).

### 3.3. Meta-analysis of open-label extension trials

**Fig. 3.**
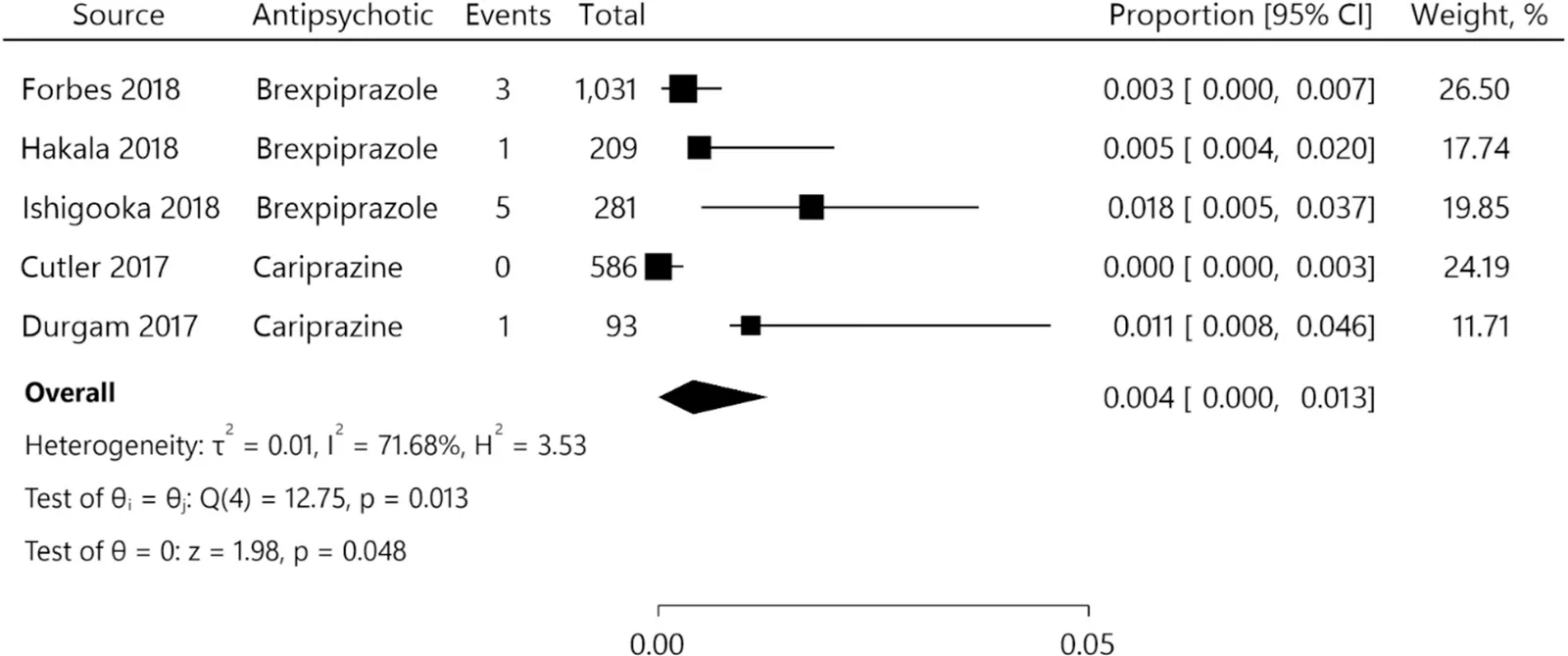
Single proportion meta-analysis of suicide deaths and attempts in open-label extension trials of dopamine partial agonists.

**Fig. 4.**
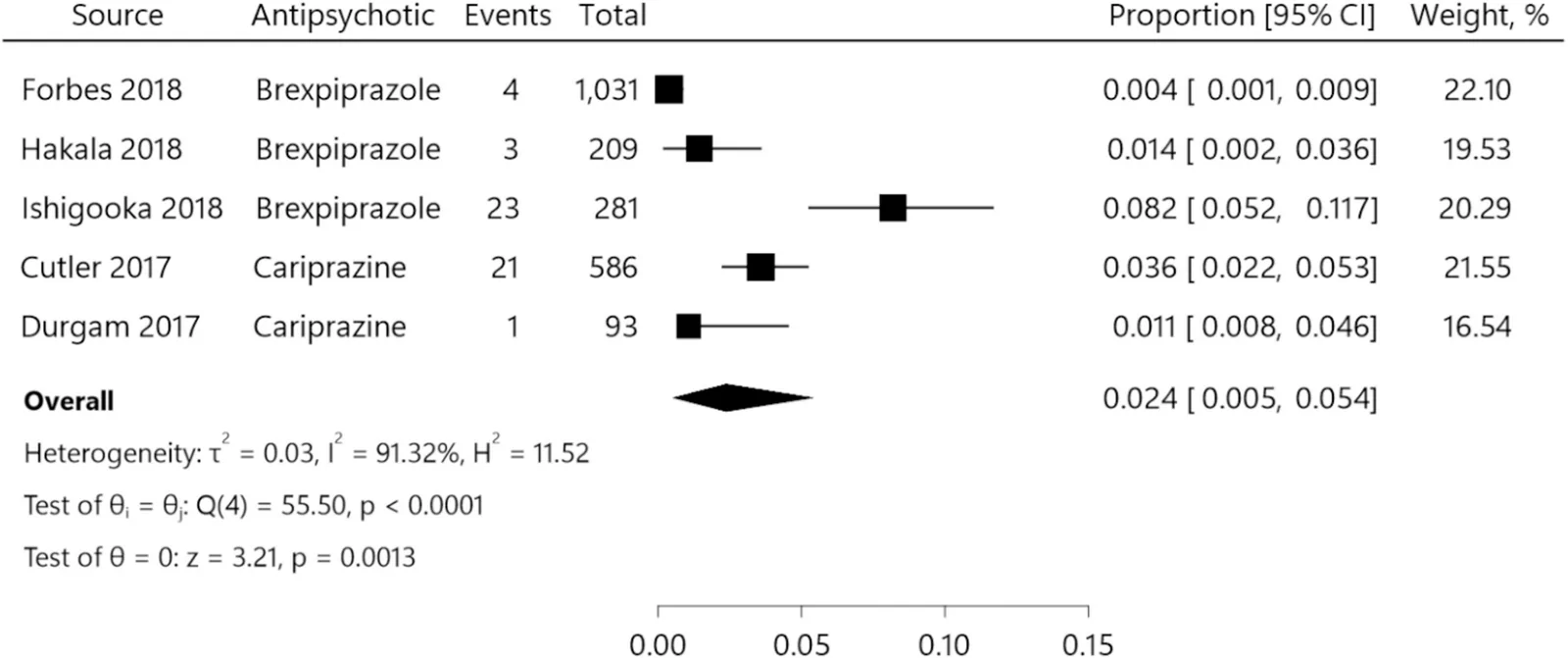
Single proportion meta-analysis of suicide deaths, attempts, and ideation in open-label extension trials of dopamine partial agonists.

Single proportion meta-analysis of OLEs that followed patients between 48 and 52 weeks showed a small but significant increase in the incidence of suicide attempts and deaths [pooled incidence = 0.004, 95% CI = 0.000–0.013, *p* = 0.048] (Fig. 3) and suicide deaths, attempts, and ideation [pooled incidence = 0.024, 95% CI = 0.005–0.054, *p* = 0.0013] (Fig. 4); however, there was marked heterogeneity across primary [*I*^2^ = 71.68%, *H*^2^ = 3.53; Q(4) = 12.75, *p* = 0.013] and secondary [*I*^2^ = 91.32%, *H*^2^ = 11.52; Q(4) = 55.50, *p* < 0.0001] suicide outcomes.

### 3.4. Publication bias

The constructed funnel plots demonstrated no qualitative evidence of asymmetry and was confirmed with Egger’s regression test for small-study effects for RCTs [*t* = 1.20, *p* = 0.25] and OLEs [*t* = 1.35, *p* = 0.27], suggesting no substantial publication bias across included studies (Supplementary Figs. S12, S13).

## 4. Discussion

Our systematic review and meta-analysis RCTs showed no significant effect of DPAs on suicidal attempts, behaviors, or ideation in persons with SCZ compared to placebo control. Subgroup analyses by antipsychotic agent and choice of comparator arm showed no significant effects on suicide outcomes. Additionally, our single proportion meta-analysis of OLEs showed a significant increase in the occurrence of suicidal attempts, behaviors, or ideation in persons with SCZ due to time at risk, however, there was high heterogeneity between studies. Based on these findings, selection of DPAs for clinical use should be driven by other considerations and not primarily by a specific goal for suicide prevention.

The lack of significant effect of DPAs on suicide risk among included RCTs may be explained by the rarity of suicide, short-term trial durations, and intentional exclusion of patients with higher suicide risk which may have limited the occurrence and capture of suicide events. The current findings appear consistent with prior, limited observational data on aripiprazole showing non-significant effects on suicidal behavior and mortality (Forte et al., 2021; Kozhevnikova et al., 2026). Despite aripiprazole being the most studied DPA, trials investigating its effects on suicide risk remain limited. Among antipsychotics, clozapine remains unique in its suicide protective effects (Meltzer et al., 2003).

Additionally, brexpiprazole and cariprazine subgroup analyses showed no increased suicide outcomes despite their higher risk of akathisia (Keks et al., 2020). However, in OLEs investigating moderate doses of brexpiprazole and cariprazine, there was a significantly increased pooled incidence of suicide events despite the exclusion of participants with higher suicide risk in four studies due to increased time at risk, but high heterogeneity across studies limits the conclusiveness of these findings. Our findings are consistent with prior research and demonstrate no conclusive evidence to support a linkage between akathisia and suicidality, but a possible increased signal influenced by high heterogeneity was observed that needs replication (Bjarke et al., 2022; Hansen, 2001; Kalniunas et al., 2021; Pompili et al., 2007; Reutfors et al., 2016). Furthermore, one non-extension open-label study of brexpiprazole and another open-label study phase of cariprazine also showed a high amount of suicide deaths, attempts, and ideation, consistent with our findings (Citrome et al., 2016; Durgam et al., 2016). These OLE findings suggest that longer trial periods may be necessary to adequately observe and evaluate suicide risk.

Only two RCTs for lumateperone were included and both trials had no suicide attempts or deaths in either study arm (Correll et al., 2020; Lieberman et al., 2016). There were an inadequate number of studies to conduct a subgroup analysis of the effects of lumateperone on suicide outcomes. Additionally, there were no eligible OLEs for lumateperone to describe longitudinal effects on suicide outcomes. The low number of studies and suicide events limits our ability to comment on the impact of lumateperone on suicide risk in SCZ. However, the potential for lumateperone to impact suicide remains interesting due to its enhancement of NMDA receptors that are implicated in suicide (Nowak et al., 1995; Sowa-Kućma et al., 2013).

Pending trials include an ongoing phase 1 randomized open label trial examining the long-acting injection formulation of brexpiprazole in patients with SCZ indicated intent to track suicide outcomes with the C-SSRS, but results are not yet publicly available (Sichuan Kelun Pharmaceutical Research Institute Co., 2024). Furthermore, our literature search identified an RCT studying JX11502MA, an investigational partial D2 and D3 agonist, that demonstrated no suicidal behavior or ideation among a small sample of healthy Chinese subjects (Yu et al., 2024). The initial pharmacokinetic and safety findings suggest the potential of JX11502MA as a favorable treatment option for SCZ.

Most of the eligible studies excluded participants that were at a higher risk for suicide (Table 1). Although RCTs are considered the gold standard for establishing causation, they have significant limitations when studying suicide due to the rare occurrence of suicide events and the more likely exclusion of participants at higher suicide risk. Effective solutions are needed to include higher risk populations in clinical research focused on suicide prevention. The emergence of alternative study designs such as target trial emulation and instrumental variable analysis may be particularly useful for suicide prevention research (Kutcher et al., 2021; Stel et al., 2013). These study designs can emulate RCTs under appropriate conditions and may overcome some logistical challenges of conducting RCTs in high-risk populations. However, limitations including potential for increased missing data and decreased data quality from lower reliability and validity compared to RCTs should be noted. Some studies have used these methods to investigate suicide outcomes in post-traumatic stress and depressive disorders, but there are no similar studies available for SCZ (Lagerberg et al., 2023; Miranda et al., 2025; Tsai et al., 2026).

### 4.1. Strengths and limitations

Strengths of the current study include use of a comprehensive multi-database article search strategy, pooling of suicide data from high quality RCTs with overall low risk of bias, employment of multiple sensitivity analyses for accuracy and bias reduction, and analysis of longer-term OLEs for more longitudinal suicide outcomes. However, we also acknowledge some limitations. First, the rare occurrence of suicide, short-term trial durations, and exclusion of patients with higher suicide risk may confound the true occurrence of suicide events. Second, the inclusion of suicidal ideation as an outcome introduces heterogeneity risk given variability in symptom reporting. Third, the high heterogeneity across OLEs with some concerns of bias illustrate considerable differences across studies and limit the generalizability of single proportion meta-analysis results. Fourth, three studies could not be included due to the lack of data availability despite reaching out to authors. These studies focused on cariprazine (no trial registration number), lumateperone (NCT02469155), and oxaripiprazole (NCT01490086) (Cantillon et al., 2017; Nakamura et al., 2016). Additionally, although our search strategy was extensive and peer-reviewed, there is a possibility that other relevant studies may have been missed.

## 5. Conclusion

Our findings demonstrate that there is no current evidence to indicate that newer DPAs causally affect suicide attempts, behaviors, or ideations for persons with SCZ in either a positive or negative way. Subgroups analyses by antipsychotic agent and comparator arm showed no significant changes to suicide risk. While there was an increased incidence of primary and secondary suicide outcomes in OLEs of brexpiprazole and cariprazine due to increased time at risk, this signal should be interpreted with caution due to high heterogeneity and requires replication in other data. Clinically, selection of DPAs for antipsychotic treatment should be driven by other considerations and not primarily by a specific goal for suicide prevention. Available evidence is limited and underscores the critical need for more benign pharmacologic alternatives that are equally effective in preventing suicide in SCZ. Future studies of rare suicide outcomes are needed that consider alternative high-quality study designs with larger sample sizes, longer term follow up, and safe to include high risk populations in clinical research focused on suicide prevention.

## Supplementary Material

Supplementary Material 1

Supplementary Material 2

Supplementary Material 3

## Figures and Tables

**Table 1:** Characteristics of included randomized controlled trials and open-label extension.

Study	NCT #	Design	Duration^a^ (weeks)	Antipsychotic	Dose range	Comparators	Total *N* (safety)	Mean age^b^ (years, SD)	Suicide assessment	Suicide exclusions
Correll 2015	NCT01396421	RCT	10	Brexpiprazole	0.25–4 mg	Placebo	636	40.2 (10.9)	C-SSRS	–
Kane 2015a*	NCT01393613	RCT	10	Brexpiprazole	1–4 mg	Placebo	674	38.5 (11.2)	C-SSRS	–
Lundbeck 2016	NCT01810380	RCT	6	Brexpiprazole	2–4 mg	Placebo Quetiapine	464	40.6 (10.8)	C-SSRS	Significant risk
Fleishhacker 2017	NCT01668797	RCT	56	Brexpiprazole	1–4 mg	Placebo	201	40.2 (10.7)	C-SSRS	Significant risk
Ishigooka 2018	NCT01451164	RCT	10	Brexpiprazole	1–4 mg	Placebo	458	44.3 (11.8)	C-SSRS	–
Durgam 2014	NCT00694707	RCT	8	Cariprazine	1.5–4 mg	Placebo Risperidone^c^	729	36.4 (10.5)	NR	Attempt or intent in past 2 years
Kane 2015b	NCT01104779	RCT	8	Cariprazine	3–9 mg	Placebo	446	36.3 (10.4)	C-SSRS	Attempt in past 2 years or significant risk
Durgam 2015	NCT01104766	RCT	8	Cariprazine	3–6 mg	Placebo Aripiprazole	617	38.5 (10.8)	C-SSRS	Attempt in past 2 years or significant risk
Durgam 2016a	NCT00404573	RCT	10	Cariprazine	1.5–12 mg	Placebo	389	41.3 (10)	NR	Intent in past 2 years
Durgam 2016b	NCT01412060	RCT	50	Cariprazine	3–9 mg	Placebo	200	38.5 (10.5)	C-SSRS	Attempt in past 2 years or significant risk
Nemeth 2017	EudraCT, 2012-005485–36	RCT	28	Cariprazine	3–6 mg	Risperidone	460	40.5 (10.9)	C-SSRS	–
Allergan Limited 2021	NCT03593213	RCT	26*	Cariprazine	3–4.5 mg	Placebo	156	41.7 (10.9)	C-SSRS	–
Lieberman 2016	NCT01499563	RCT	6	Lumateperone	42–84 mg	Placebo	334	36.4 (10.8)	C-SSRS	Ideation and behavior
Correll 2020	NCT02282761	RCT	6	Lumateperone	28–42 mg	Placebo	449	42.4 (10.2)	C-SSRS	Ideation and behavior
Cantillon 2018	None	RCT	2.5	Oxaripiprazole	10–100 mg	Placebo	32	43.9 (9.5)	C-SSRS	–
Forbes 2018^d^	NCT01397786	OLE	56	Brexpiprazole	1–4 mg	–	1031	40 (11.1)	C-SSRS	Significant risk
Hakala 2018^e^	NCT01810783	OLE	52*	Brexpiprazole	1–4 mg	–	209	40.9 (11.4)	C-SSRS	Significant risk
Ishigooka 2018^f^	NCT01456897	OLE	56	Brexpiprazole	1–4 mg	–	281	44.4 (14)	C-SSRS	–
Cutler 2017^g^	None	OLE	52	Cariprazine	3–9 mg	–	586	39.1 (10.8)	C-SSRS	Attempt in past 2 years or significant risk
Durgam 2017^h^	NCT00839852	OLE	52	Cariprazine	1.5–4.5 mg	–	93	34.4 (10.1)	STS	Significant risk

**Key:** RCT = randomized controlled trial, OLE = open-label extension trial, NR = not reported, C-SSRS = Columbia Suicide Severity Rating Scale, STS = Suicidality Tracking Scale.

* Follow up phase not reported.

a Total trial duration includes active trial and safety follow up phases.

b The mean ages and standard deviations were averaged across study arms if values for total sample size were not available.

c Sensitivity analysis only.

d Extension study of Correll 2015 (NCT01396421), Kane 2015a (NCT01393613), and Fleishhacker 2017 (NCT01668797).

e Extension study of Lundbeck 2016 (NCT01810380).

f Extension study of Ishigooka 2018 (NCT01451164).

g Extension study of Kane 2015b (NCT01104779) and Durgam 2015 (NCT01104766).

h Extension study of Durgam 2014 (NCT00694707).

## Data Availability

All data are available in the manuscript, figures, tables, and supplementary files.

## Appendix A. Supplementary data

Supplementary data to this article can be found online at https://doi.org/10.1016/j.schres.2026.09.006.
