# Supplementary material for: Effect of dopamine partial agonist antipsychotics on suicide risk in persons with schizophrenia and schizoaffective disorders: A systematic review and meta-analysis
Source: Schizophr Res. Author manuscript; Available in PMC 2026 Sep 18. (PMC13583873; doi:10.1016/j.schres.2026.09.006)
Supplement: Supplementary file 1 [file NIHMS2209796-supplement-Supplementary_Material_2.pdf]

NIHR | National Institute for Health and Care Research

PROSPERO
International prospective register of systematic reviews

Effect of Third Generation Antipsychotics on Suicide Risk in Persons with Schizophrenia Spectrum Disorders

Jeff Jin, Charlotte Winkler, Heather Blunt, Natalie Riblet

Review methods were amended after registration. Please see the revision notes and previous versions for detail.

To enable PROSPERO to focus on COVID-19 submissions, this registration record has undergone basic automated checks for eligibility and is published exactly as submitted. PROSPERO has never provided peer review, and usual checking by the PROSPERO team does not endorse content. Therefore, automatically published records should be treated as any other PROSPERO registration. Further detail is provided here.

Citation 1 change
Jeff Jin, Charlotte Winkler, Heather Blunt, Natalie Riblet. Effect of Third Generation Antipsychotics on Suicide Risk in Persons with Schizophrenia Spectrum Disorders. PROSPERO 2023 CRD42023449336. Available from https://www.crd.york.ac.uk/PROSPERO/view/CRD42023449336.

REVIEW TITLE AND BASIC DETAILS

Review title 1 change

Effect of Third Generation Antipsychotics on Suicide Risk in Persons with Schizophrenia Spectrum Disorders

Review objectives

In persons with schizophrenia and schizoaffective disorder, what effects do newer third generation antipsychotics have on suicidal risk?

- P - Schizophrenia Spectrum Illness
- I - 3rd generation antipsychotics (cariprazine, lumateperone, brexpiprazole, oxaripiprazole)
- C - Placebo vs. other antipsychotics
- O - Suicide attempts and completions

Keywords

Antipsychotics; Meta-analysis; Psychotic disorders; Suicide; Schizophrenia; Schizoaffective

SEARCHING AND SCREENING

Searches 1 change

- Databases: PubMed/MEDLINE, PsycINFO (EBSCO), Cochrane Central Register of Controlled Trials (Wiley), Scopus, Europe PMC (preprints), ClinicalTrials.gov
- Search Dates: Inception to February 9th, 2026

Study design 1 change

Both randomized and nonrandomized study types will be included.

Included

Randomized Controlled Trials, Open Label Extension Trials

Excluded

The following studies will be excluded:

- Non-Human subject studies
- Non-Adult subject studies
- Non-English publications
- Wrong study design (not randomized controlled trial or open label extension trial)
- Wrong diagnosis (not schizophrenia or schizoaffective disorder)
- Wrong intervention (not third-generation antipsychotic)
- Studies focused on aripiprazole
- Studies that do not report data on suicide outcomes (deaths, attempts, ideation)

Link to search strategy 1 change

A full search strategy has been uploaded to PROSPERO. The PDF may be accessed through this link https://www.crd.york.ac.uk/PROSPEROFILES/58bdfce89ddb60709eab11d7a26a51e8.pdf.

ELIGIBILITY CRITERIA

Condition or domain being studied

Schizophrenia; Schizoaffective Disorder; Schizophreniform Disorder; Schizophrenic Related Disorders; Suicide; Suicidal Behavior; Suicidal Ideation

- Schizophrenia spectrum illness as defined by the National Institute of Mental Health and Diagnostic and Statistical Manual of Mental Disorders, fifth edition (DSM-V) as diagnoses of schizophrenia, schizoaffective disorder, or an unspecified schizophrenia spectrum/other psychotic disorder.
- Schizophrenia - a mental disorder characterized by disruptions in thought processes, perceptions, emotional responsiveness, and social interactions
- Schizoaffective disorder - a chronic mental health condition characterized primarily by symptoms of schizophrenia, such as hallucinations or delusions, and symptoms of a mood disorder, such as mania and depression.

- Unspecified schizophrenia spectrum/other psychotic disorder - a mental health condition characterized by symptoms of a schizophrenia spectrum and other psychotic disorder that cause clinically significant distress or impairment in social, occupational, or other important areas of functioning predominate but do not meet the full criteria for any of the disorders in the schizophrenia spectrum and other psychotic disorders class

Population

Included

- Inclusion: Patients with schizophrenia and related psychotic spectrum illnesses (as diagnosed by any recognized diagnostic criteria for schizophrenia, schizoaffective disorder, schizophreniform, or an unspecified schizophrenia spectrum/other psychotic disorder)
- Exclusion: Non-schizophrenia spectrum illness population

Intervention(s) or exposure(s)

Included

- Third generation antipsychotics - a class of psychotropic medication used to treat psychosis that is characterized by a common pharmacodynamic feature of partial dopamine D2 agonism
- Current medications of this class include oxaripiprazole, brexpiprazole, lumateperone, and cariprazine

Excluded
Aripiprazole has been excluded to focus the meta-analysis on newer agents

Comparator(s) or control(s)

Included

- Placebo
- Non-third generation antipsychotics (treatment as usual)

Context

Studies in hospital, partial hospitalization, inpatient, and outpatient psychiatric care settings will be included.

OUTCOMES TO BE ANALYSED

Main outcomes

- Primary Outcome: suicide deaths and attempts
- Secondary Outcome: suicide deaths, attempts, and ideation
- Instruments: Validated measurements to assess suicide risk (such as the Columbia Suicide Severity Rating Scale)
- Time points: Study dependent
- Effect measure: Risk Ratio

Additional outcomes

Not applicable

DATA COLLECTION PROCESS

Data extraction (selection and coding)

- Study selection: Two reviewers will apply the eligibility criteria by independently screening records for inclusion and cross-checking selection results after screening completion. Both researchers will be blinded to the other's decisions during the selection process. Disagreements will be formally discussed and resolved by consensus. In the case a disagreement cannot be resolved, a clinically-informed third party will be consulted for resolution by majority. Rayyan QCRI is the current preferred mechanism for screening decision records.
- Data extraction: Information about study design, participant demographics, intervention type, suicide outcome, and study results will be extracted by comprehensive and in-depth article review after the screening process. Two researchers will be extracting the data while a third will be reviewing the extracted data for accuracy. Disagreements will be formally discussed and resolved by consensus. In the case a disagreement cannot be resolved, a clinically-informed third party will be consulted for resolution by majority. Missing data will be re-visited based on appearance. Data will be recorded in a shared protected-spreadsheet.

Risk of bias (quality) assessment

Two review authors will independently assess the risk of bias of included studies by using the considering the followingcharacteristics, as recommended by the International Cochrane Collaboration:

- Randomization sequence generation
- Treatment allocation concealment
- Blinding
- Completeness of outcome data
- Selective outcome reporting
- Other sources of bias

The revised Cochrane Risk-Of-Bias tool for randomized trials (RoB2) will be used. Disagreements will be formally discussed and resolved by consensus. In the case a disagreement cannot be resolved, a clinically-informed third party will be consulted for resolution by majority. The level of risk of bias in each of these domains will be presented separately for each study in tables in the final review publication.

PLANNED DATA SYNTHESIS

Strategy for data synthesis

- Software: Stata SE18

- Statistical Significance: p <0.05
- Effects Size Estimates: Risk Ratios, 95% Confidence Intervals
- For randomized controlled trials, a random-effects meta-analysis will be used
- For open label extension studies, a single proportion meta-analysis will be used
- For study arms with zero events, a continuity correction of 0.5 was added to prevent mathematical impossibility (standard in Stata SE18)
- Study heterogeneity will be explored statistically (defined as I-squared > 50% or $p < 0.1$)
- Publication bias will be assessed with funnel plots and Egger's tests

Analysis of subgroups or subsets

- Sensitivity analyses will be conducted to exclude randomized controlled trials with "some concerns" or "high risk" of bias
- Subgroup analyses will be conducted for randomized controlled trials to explore the impact of dose, drug type, and choice of comparator arm on suicide outcomes

REVIEW AFFILIATION, FUNDING AND PEER REVIEW

Review team members

Dr Jeff Jin (review guarantor and contact) Dartmouth-Hitchcock Medical Center. United States of America.

No conflict of interest declared.

Dr Charlotte Winkler. Dartmouth-Hitchcock Medical Center. United States of America.

No conflict of interest declared.

Mrs Heather Blunt. Dartmouth-Hitchcock Medical Center. United States of America.

No conflict of interest declared.

Dr Natalie Riblet. Dartmouth-Hitchcock Medical Center. United States of America.

No conflict of interest declared.

Named contact

Dr Jeff Jin (jeff.w.jin@hitchcock.org). Dartmouth-Hitchcock Medical Center. United States of America.

Review affiliation

Dartmouth-Hitchcock Medical Center

Funding source

Not applicable

TIMELINE OF THE REVIEW

Review timeline 1 change

Start date: 14 August 2023. End date: 9 February 2026.

Date of first submission to PROSPERO
09 August 2023

Date of registration in PROSPERO
21 August 2023

CURRENT REVIEW STAGE

Publication of review results 1 change
Results of the review will be published in English.

Journal publication
Not yet published in a journal but will be in future.

Stage of the review at this submission

Review stage	Started	Completed
Pilot work	✓	✓
Formal searching/study identification	✓	✓
Screening search results against inclusion criteria	✓	✓
Data extraction or receipt of IPD	✓	✓
Risk of bias/quality assessment	✓	✓
Data synthesis	✓	✓

Review status
The review is completed.

ADDITIONAL INFORMATION

PROSPERO version history 1 change

- Version 4.0, published 11 Feb 2026
- Version 3.0, published 04 Feb 2026
- Version 2.0, published 20 Nov 2025
- Version 1.4, published 20 Jan 2025
- Version 1.3, published 12 Feb 2024
- Version 1.2, published 05 Dec 2023
- Version 1.1, published 21 Aug 2023
- Version 1.0, published 21 Aug 2023

Review conflict of interest
None known

Country
United States of America

Medical Subject Headings

Antipsychotic Agents; Humans; Schizophrenia; Suicidal Ideation; Suicide, Attempted; brexpiprazole; cariprazine; lumateperone; Suicide, Completed

Revision note 1 change

Inclusion of updated search strategies to include articles from December 1st, 2023 until February 9th, 2026.

Disclaimer

The content of this record displays the information provided by the review team. PROSPERO does not peer review registration records or endorse their content.

PROSPERO accepts and posts the information provided in good faith; responsibility for record content rests with the review team. The guarantor for this record has affirmed that the information provided is truthful and that they understand that deliberate provision of inaccurate information may be construed as scientific misconduct.

PROSPERO does not accept any liability for the content provided in this record or for its use. Readers use the information provided in this record at their own risk.

Any enquiries about the record should be referred to the named review contact
