# Supplementary material for: Effect of dopamine partial agonist antipsychotics on suicide risk in persons with schizophrenia and schizoaffective disorders: A systematic review and meta-analysis
Source: Schizophr Res. Author manuscript; Available in PMC 2026 Sep 18. (PMC13583873; doi:10.1016/j.schres.2026.09.006)
Supplement: Supplementary file 2 [file NIHMS2209796-supplement-Supplementary_Material_3.pdf]

Search Strategies
Effect of Third Generation Antipsychotics on Suicide Risk in Persons with Schizophrenia and Schizoaffective Disorder

Databases

Database	Platform	Years covered	Dates conducted	# results
Medline	PubMed	1946 – 2026	December 1, 2023<br>February 9, 2026	1569
APA PsycInfo	EBSCO	1806 – 2026	December 1, 2023<br>February 9, 2026	473
Cochrane Central Register of Controlled Trials	Wiley	Feb 2026	December 1, 2023<br>February 9, 2026	837
EuropePMC	EuropePMC.org	2018 – 2026	December 1, 2023<br>February 9, 2026	51
Scopus	Elsevier	1788 – 2026	December 1, 2023<br>February 9, 2026	2144
Total				5074
Total with duplicates removed				**3558**

Search Strategies

Initial search performed on December 1, 2023 (inception to December 1, 2023)
Searched by Heather Blunt, Dartmouth Biomedical Libraries
Peer reviewed by Elaina Vitale, Dartmouth Biomedical Libraries

Updated search performed using peer-reviewed search design on February 9, 2026 (to include articles from December 1, 2023, to February 9, 2026)
Searched by Jeff Jin, Dartmouth-Hitchcock Medical Center

PubMed

Search	Query	Results
#5	Search: #1 AND #2 AND #3 AND #4	1569
#4	Search: ((randomized controlled trial[pt] OR controlled clinical trial[pt] OR randomized[tiab] OR randomised[tiab] OR placebo[tiab] OR drug therapy[sh] OR randomly[tiab] OR trial[tiab] OR groups[tiab]) NOT (animals[mh] NOT humans[mh]))	5,895,831

#3	Search: "Schizophrenia Spectrum and Other Psychotic Disorders"[Mesh] OR "Schizoid Personality Disorder"[Mesh] OR "Schizotypal Personality Disorder"[Mesh] OR schizo*[tiab] OR Psychotic[tiab] OR Psychosis[tiab] OR Psychoses[tiab] OR deficit syndrome[tiab]	271,930
#2	Search: "Aripiprazole"[Mesh] OR "brexpiprazole" [Supplementary Concept] OR "cariprazine" [Supplementary Concept] OR "lumateperone" [Supplementary Concept] OR abilify[tiab] OR aripiprazole [tiab] OR aristada[tiab] OR brexpiprazole[tiab] OR brilaroxazine[tiab] OR caplyta[tiab] OR cariprazine[tiab] OR lumateperone[tiab] OR oxaripiprazole[tiab] OR reagila[tiab] OR rexulti[tiab] OR RP5063[tiab] OR vraylar[tiab] OR ((3rd generation[tiab] OR third generation[tiab]) AND antipsychotic*[tiab]) OR ((D2[tiab] OR "D(2)"[tiab] OR D3[tiab] OR "D(3)"[tiab] OR dopamine receptor[tiab]) AND agonis*[tiab] AND partial[tiab])	8,038
#1	Search: "Suicide"[Mesh] OR "adverse effects" [Subheading:NoExp] OR "Safety"[Mesh] OR suicid*[tiab] OR adverse event*[tiab] OR adverse effect*[tiab] OR Safe*[tiab]	3,722,576

Cochrane Central Register of Controlled Trials (Wiley)

ID	Search	Hits
#1	(suicid* OR (adverse NEXT event*) OR (adverse NEXT effect*) OR safe*):ti,ab,kw (Word variations have been searched)	620,697
#2	(abilify OR aripiprazole OR aristada OR brexpiprazole OR brilaroxazine OR caplyta OR cariprazine OR lumateperone OR oxaripiprazole OR reagila OR rexulti OR RP5063 OR vraylar OR (("3rd generation" OR "third generation") AND antipsychotic*) OR ((D2 OR "D(2)" OR D3 OR "D(3)" OR "dopamine receptor") AND agonis* AND partial)):ti,ab,kw (Word variations have been searched)	2,609
#3	(schizo* OR psychoses OR psychosis OR psychotic OR "deficit syndrome"):ti,ab,kw (Word variations have been searched)	30,094
#4	#1 AND #2 AND #3	865
#5	#1 AND #2 AND #3 in Trials	837

PsycInfo (Ebsco)

**#**	**Query**	**Results**

S5	S1 AND S2 AND S3 AND S4	473
S4	(DE "Randomized Controlled Trials" OR DE "Treatment Effectiveness Evaluation") OR (random* OR control* OR placebo)	1,104,854
S3	(DE "Acute Schizophrenia" OR DE "Affective Psychosis" OR DE "Brief Psychotic Disorder" OR DE "Capgras Syndrome" OR DE "Catatonic Schizophrenia" OR DE "Childhood Onset Psychosis" OR DE "Childhood Onset Schizophrenia" OR DE "Chronic Psychosis" OR DE "Delusional Disorder" OR DE "Paranoid Psychosis" OR DE "Paranoid Schizophrenia" OR DE "Postpartum Psychosis" OR DE "Process Schizophrenia" OR DE "Psychosis" OR DE "Reactive Psychosis" OR DE "Schizoid Personality Disorder" OR DE "Schizophrenia" OR DE "Schizophrenia (Disorganized Type)" OR DE "Schizophreniform Disorder" OR DE "Schizotypal Personality Disorder" OR DE "Undifferentiated Schizophrenia") OR (schizo* OR psychoses OR psychosis OR psychotic OR "deficit syndrome")	298,604
S2	DE "Aripiprazole" OR (abilify OR aripiprazole OR aristada OR brexpiprazole OR brilaroxazine OR caplyta OR cariprazine OR lumateperone OR oxaripiprazole OR reagila OR rexulti OR RP5063 OR vraylar OR (("3rd generation" OR "third generation") AND antipsychotic*) OR ((D2 OR "D(2)" OR D3 OR "D(3)" OR "dopamine receptor") AND agonis* AND partial))	4,269
S1	(DE "Attempted Suicide" OR DE "Military Suicide" OR DE "Suicidal Behavior" OR DE "Suicidal Ideation" OR DE "Suicidality" OR DE "Suicide" OR DE "Youth Suicide" OR DE "Patient Safety" OR DE "Safety") OR (suicid* OR "adverse event*" OR "adverse effect*" OR Safe*)	302,937

Scopus

	Search Terms	Results
5	(TITLE-ABS-KEY (suicid* OR "adverse event*" OR "adverse effect*" OR safe*)) AND (TITLE-ABS-KEY (abilify OR aripiprazole OR aristada OR brexpiprazole OR brilaroxazine OR caplyta OR cariprazine OR lumateperone OR oxaripiprazole OR reagila OR rexulti OR rp5063 OR vraylar OR (("3rd generation" OR "third generation") AND antipsychotic*) OR ((d2 OR "D(2)" OR d3 OR "D(3)" OR "dopamine receptor") AND agonis* AND partial))) AND (TITLE-ABS-KEY (schizo* OR psychoses OR psychosis OR psychotic OR "deficit syndrome")) AND ((INDEXTERMS ("randomized controlled trial" OR "controlled clinical trial" OR "drug therapy")) OR (TITLE-ABS ((randomized OR randomised OR placebo OR randomly OR trial OR groups))) OR (AUTHKEY (random* OR rct OR trial)))	2,144 document results
4	(INDEXTERMS ("randomized controlled trial" OR "controlled clinical trial" OR "drug therapy")) OR (TITLE-ABS ((randomized OR randomised OR placebo OR randomly OR trial OR groups))) OR (AUTHKEY (random* OR rct OR trial))	13,038,618 document results

3	TITLE-ABS-KEY (schizo* OR psychoses OR psychosis OR psychotic OR "deficit syndrome")	407,038 document results
2	TITLE-ABS-KEY (abilify OR aripiprazole OR aristada OR brexpiprazole OR brilaroxazine OR caplyta OR cariprazine OR lumateperone OR oxaripiprazole OR reagila OR rexulti OR rp5063 OR vraylar OR (("3rd generation" OR "third generation") AND antipsychotic*) OR ((d2 OR "D(2)" OR d3 OR "D(3)" OR "dopamine receptor") AND agonis* AND partial))	23,086 document results
1	TITLE-ABS-KEY (suicid* OR "adverse event*" OR "adverse effect*" OR safe*)	4,379,152 document results

Europe PMC (preprints)

(abilify OR aripiprazole OR aristada OR brexpiprazole OR brilaroxazine OR caplyta OR cariprazine OR lumateperone OR oxaripiprazole OR reagila OR rexulti OR rp5063 OR vraylar OR (("3rd generation" OR "third generation") AND antipsychotic*) OR ((d2 OR "D(2)" OR d3 OR "D(3)" OR "dopamine receptor") AND agonis* AND partial)) AND (suicid* OR "adverse event*" OR "adverse effect*" OR safe*) AND (schizo* OR psychoses OR psychosis OR psychotic OR "deficit syndrome") AND (SRC:PPR)

51 results
