# Supplementary material for: Effect of dopamine partial agonist antipsychotics on suicide risk in persons with schizophrenia and schizoaffective disorders: A systematic review and meta-analysis
Source: Schizophr Res. Author manuscript; Available in PMC 2026 Sep 18. (PMC13583873; doi:10.1016/j.schres.2026.09.006)
Supplement: Supplementary file 3 [file NIHMS2209796-supplement-Supplementary_Material_1.docx]

**Supplementary Materials**

Suicide Risk of Third-Generation Antipsychotics in Persons with Schizophrenia and Schizoaffective Disorders: A Systematic Review and Meta-Analysis

Jin JW^1^, Winkler CJ^1^, Blunt HB^2^, Riblet NB^3,4,5^

^1^ Dartmouth-Hitchcock Medical Center – Department of Psychiatry

^2^ Biomedical Libraries, Dartmouth College, Hanover, New Hampshire, USA

^3^ Mental Health, White River Junction VA Healthcare System, White River Junction, VT 05009, USA

^4^ Psychiatry, Geisel School of Medicine at Dartmouth College, Hanover, NH 03755, USA

^5^ Dartmouth Institute, Geisel School of Medicine at Dartmouth College, Hanover, NH 03755, USA

**Inclusion Criteria**

1. Human Subjects greater than 18 years of age
2. English Language
3. Randomized Controlled Trial or Open Label Extension Trial Design
4. Diagnostic and Statistical Manual of Mental Disorders Fourth Edition (DSM-IV) diagnosis of schizophrenia, schizoaffective disorder, schizophreniform disorder, unspecified psychosis
5. Third generation antipsychotic intervention (oxaripiprazole, brexpiprazole, cariprazine, lumateperone, or another D2/D3 partial agonist)
6. Reported data on suicide outcomes (deaths, attempts, and ideation)

**Exclusion Criteria**

1. Cannabis or substance-induced psychosis only diagnoses were excluded
2. Prodromal psychosis states including early phase psychosis, sub-clinical psychosis, and clinical high-risk for psychosis were excluded
3. Self-reported psychosis diagnoses were excluded
4. Studies focused on aripiprazole were excluded
5. Data on non-suicidal self-injurious behavior were excluded from analysis

**Supplementary Figure S1.** PRISMA Flow Diagram for study inclusion.

**Supplementary Table S1.** Risk of Bias Assessment for Randomized Controlled Trials by Domain

**Supplementary Table S2.** Risk of Bias Assessment for Open Label Extension Trials by Domain

*****No information for D1 due to lack of randomization in open label trials

**Supplementary Figure S2.** Risk of Bias of Included Randomized Controlled Trials.

**Supplementary Figure S3.** Risk of Bias of Included Open-Label Extensions Trials.

*****No information for randomization bias due to lack of randomization in open label trials

**Supplementary Figure S4.** Sensitivity Analysis of Suicide Deaths and Attempts in Randomized Controlled Trials

**Supplementary Figure S5.** Sensitivity Analysis of Suicide Deaths, Attempts, and Ideation in Randomized Controlled Trials

**Supplementary Figure S6.** Subgroup Analysis of Suicide Deaths, Attempts, and Ideation in Randomized Controlled Trials by Antipsychotic Agent

**Supplementary Figure S7.** Sensitivity Analysis of Subgroup Analysis of Suicide Deaths, Attempts, and Ideation in Randomized Controlled Trials by Antipsychotic Agent

**Supplementary Figure S8.** Subgroup Analysis of Suicide Deaths and Attempts in Randomized Controlled Trials by Comparator

**Supplementary Figure S9.** Sensitivity Analysis of Subgroup Analysis of Suicide Deaths and Attempts in Randomized Controlled Trials by Comparator

**Supplementary Figure S10.** Subgroup Analysis of Suicide Deaths, Attempts, and Ideation in Randomized Controlled Trials by Comparator

**Supplementary Figure S11.** Sensitivity Analysis of Subgroup Analysis of Suicide Deaths, Attempts, and Ideation in Randomized Controlled Trials by Comparator

**Supplementary Figure S12.** Overall Funnel Plot for Included Randomized Controlled Trials

**Supplementary Figure S13.** Overall Funnel Plot for Included Open Label Extension Trials

**Supplementary Table S3.** Results of sensitivity analysis using Poisson or Negative Binomial Regression Models.

|  | | **Drug PY** | | **Placebo PY** | |  |  |
| --- | --- | --- | --- | --- | --- | --- | --- |
|  | **Total Studies** | **Events** | **Total PY** | **Events** | **Total PY** | **Model Type** | **IRR (95% CI)** |
| ***Suicide deaths or attempts in randomized controlled trials*** | | | | | | | |
| Any DPA  (main analysis) | 14 | 3 | 781.08 | 1 | 466.15 | Poisson | 1.67  (0.17-16.33) |
| Any DPA  (sensitivity analysis based on quality) | 12 | 2 | 712.27 | 1 | 421.08 | Poisson | 1.03  (0.09-11.76) |
| ***Suicide deaths, attempts, or ideation in randomized controlled trials*** | | | | | | | |
| Any DPA  (main analysis) | 14 | 18 | 781.08 | 14 | 466.15 | NB | 0.76  (0.20-2.96) |
| Any DPA  (sensitivity analysis based on quality) | 12 | 17 | 712.27 | 14 | 421.08 | NB | 0.67  (0.16-2.74) |
| Brexiprazole  (main analysis) | 5 | 4 | 366.19 | 2 | 222.88 | NB | 1.22  (0.22-6.65) |
| Brexiprazole (sensitivity analysis based on quality) | 4 | 3 | 348.89 | 2 | 204.31 | NB | 0.88  (0.15-5.26) |
| Cariprazine  (main analysis) | 6 | 10 | 359.85 | 10 | 215.88 | NB | 0.54  (0.07-4.49) |
| Cariprazine (sensitivity analysis based on quality) | 5 | 10 | 308.35 | 10 | 189.38 | NB | 0.54  (0.07 –4.21) |

*Key: CI = Confidence Interval; DPA = dopamine partial agonist antipsychotic; IRR = Incidence Rate Ratio; NB = Negative binomial; PY = Person-years*

**Supplementary Table S4**. Qualitative findings regarding the relationship between antipsychotic dose and suicide-related outcomes.

|  |  | **Drug** | | **Placebo** | |
| --- | --- | --- | --- | --- | --- |
|  | **Total Studies** | **Events** | **Total** | **Events** | **Total** |
| ***Suicide deaths and attempts in randomized controlled trials*** | | | | | |
| Brexiprazole | 5 | 2 | 1,518 | 1 | 745 |
| Brexiprazole < 2 mg | 3 | 0 | 322 | 1 | 480 |
| Brexiprazole ≥ 2 mg | 4 | 1 | 1,099 | 1 | 641 |
| Cariprazine | 6 | 1 | 1,512 | 0 | 732 |
| Cariprazine < 6 mg | 5 | 0 | 973 | 0 | 633 |
| Cariprazine ≥ 6 mg | 3 | 1 | 438 | 0 | 429 |
| ***Suicide deaths, attempts and ideation in randomized controlled trials*** | | | | | |
| Brexiprazole ≥ 2 mg | 4 | 2 | 1,099 | 1 | 641 |
| Cariprazine < 6 mg | 5 | 3 | 973 | 8 | 633 |
| Cariprazine ≥ 6 mg | 3 | 6 | 438 | 8 | 429 |

**References for Included Randomized Trials of Brexpiprazole.**

| **N** | **Citation** | **Registry Number** |
| --- | --- | --- |
| 1 | Correll CU, Skuban A, Ouyang J, et al. Efficacy and safety of Brexpiprazole for the treatment of acute schizophrenia: A 6-week randomized, double-blind, placebo-controlled trial. *Am J Psychiatry* 2015; 172(9): 870-880. | NCT01396421 |
| 2 | Fleischhacker WW, Hobart M, Ouyang J, et al. Efficacy and safety of Brexpiprazole (OPC-34712) as maintenance treatment in adults with schizophrenia: A randomized, double-blind, placebo-controlled study. International Journal of Neuropsychopharmacology 2017; 20(1): 11-21. | NCT01668797 |
| 3 | Ishigooka J, Iwashita S, Tadori Y. Efficacy and safety of Brexiprazole for the treatment of acute schizophrenia in Japan: A 6-week, randomized double-blind, placebo-controlled study. *Psychiatry and Clinical Neurosciences* 2018; 72: 692-700. | NCT01451164 |
| 4 | Kane JM, Skuban A, Ouyang J, et al. A multicenter, randomized, double-blind, controlled phase 3 trial of a fixed-dose Brexiprazole for the treatment of adults with acute schizophrenia. *Schizophrenia Research* 2015; 164: 127-135. | NCT01393613 |
| 5 | Lundbeck. Clinical trial results: Interventional, randomised, double-blind, parallel-group, placebo-controlled, active-reference, flexible-dose study of Brexiprazole in patients with acute schizophrenia. EudraCT number 2012-002252-17. EU-CTR publication date July 14, 2016. | NCT01810380 |

**References for Included Randomized Trials of Cariprazine**

| **N** | **Citation** | **Registry Number** |
| --- | --- | --- |
| 1 | Durgam S, Starace A, Li D, et al. An evaluation of the safety and efficacy of Cariprazine in patients with acute exacerbation of schizophrenia: A phase II, randomized clinical trial. *Schizophrenia Research* 2014; 152: 450-457. | NCT00694707 |
| 2 | Durgam S, Cutler AJ, Lu K, et al. Cariprazine in acute exacerbation of schizophrenia: A fixed-dose, phase 3, randomized, double-blind, placebo- and active-controlled trial. *J Clin Psychiatry* 2015; 76(12):e1574-e1582. | NCT01104766 |
| 3 | Durgam S, Earley W, Li R, et al. Long-term Cariprazine treatment for the prevention of relapse in patients with schizophrenia: A randomized, double-blind, placebo-controlled trial. *Schizophrenia Research* 2016; 176: 264-271. | NCT01412060 |
| 4 | Durgam S, Litman RE, Papadakis K, et al. Cariprazine in the treatment of schizophrenia: A proof-of-concept trial. *International Clinical Psychopharmacology* 2016; 31: 61-68. | NCT00404573 |
| 5 | Kane JM, Zukin S, Wang Y, et al. Efficacy and safety of Cariprazine in acute exacerbation of schizophrenia: Results from an international, phase III clinical trial. *J Clin Psychopharmacol* 2015; 35: 367-373. | NCT01104779 |
| 6 | Nemeth G, Laszlovsky I, Czobor P, et al. Carpiprazine versus risperidone monotherapy for treatment of predominant negative symptoms in patients with schizophrenia: A randomised, double-blind, controlled trial. *Lancet* 2017; 389: 1103-1113. | EudraCT, number 2012-005485-36 |
| 7 | Allergan, AbbVie. Clinical trial results: Clinical Trial Evaluating the Efficacy, Safety, and Tolerability of Cariprazine in a Dose-Reduction Paradigm in the Prevention of Relapse in Participants with Schizophrenia. EudraCT number 2012-002252-17. EU-CTR publication date July 14, 2016. | NCT03593213  EudraCT, number 2017-000818-34 |

**References for Included Randomized Trials of Lumateperone**

| **N** | **Citation** | **Registry Number** |
| --- | --- | --- |
| 1 | Lieberman JA, Davis RE, Correll CU, et al. ITI-007 for the Treatment of Schizophrenia: A 4-Week Randomized, Double-Blind, Controlled Trial. Biol Psychiatry. 2016 Jun 15;79(12):952-61. | NCT01499563 |
| 2 | Correll CU, Davis RE, Weingart M, et al. Efficacy and safety of Lumateperone for treatment of schizophrenia: A randomized clinical trial. *JAMA Psychiatry* 2020; 77(4): 349-358. | NCT02282761 |

**References for Included Randomized Trials of Oxaripiprazole.**

| **N** | **Citation** | **Registry Number** |
| --- | --- | --- |
| 1 | Cantillon M, Ings R, Bhat L. Initial clinical experience of RP5063 following single doses in normal healthy volunteers and multiple doses in patients with stable schizophrenia. *Clin Transl Sci* 2018; 11: 387-396. | None |

**References for Open Label Studies.**

| **N** | **Citation** | **Registry Number** |
| --- | --- | --- |
| 1 | Forbes A, Hobart M, Ouyang J, et al. A long-term, open-label study to evaluate the safety and tolerability of Brexpiprazole as maintenance treatment in adults with schizophrenia. Interrnational Journal of Neuropsychopharmacology 2018; 21(5): 433-441. | NCT01397786 (Extension study of NCT01396421, NCT01393613, and NCT01668797) |
| 2 | Hakala M. Gislum M, Skuban A, Meehan S. 07.5. Long-term safety and tolerability of Brexapiprazole in patients with schizophrenia. 2018 Abstracts for the Sixth Biennial SIRS Conference Oral Session: Pharmacology. S94-S95. | NCT01810783 (Extension study of NCT01810380) |
| 3 | Ishigooka J, Iwashita S, Tadori Y. Long-term safety and effectiveness of Brexiprazole in Japanese patients with schizophrenia: A 52-week, open-label study. *Psychiatry and Clinical Neurosciences* 2018; 72: 445-453. | NCT01456897 (Extension study of NCT01451164) |
| 4 | Cutler AJ, Durgam S, Wang Y, et al. Evaluation of the long-term safety and tolerability of Cariprazine in patients with schizophrenia: Results from a 1-year open-label study. *CNS Spectrums* 2018; 23: 39-50. | NCT01104792 (Extension of NCT01104779 and NCT01104766) |
| 5 | Durgam S, Greenberg WM, Li D, et al. Safety and tolerability of Cariprazine in the long-term treatment of schizophrenia: Results from a 48-week, single-arm, open-label extension study. *Psychopharmacology* 2017; 234: 199-209. | NCT00839852 (Extension study of NCT00694707) |
